# Report to the Surgeon General on an Improved Method of Photographing Histological Preparations by Sunlight

**Published:** 1871-12

**Authors:** 


*Report to the Surgeon General on an Improved Method of Photographing Histological Preparations by Sunlight.* By Assistant Surgeon J. J. Woodward, U. S. Army, Washington, 1871.

In connection with a complete description of the processes adopted, we have received specimens of what may be accomplished. They very beautifully represent the following objects:

Striated muscular fibres of mouse. Magnified 500 diameters.

Small artery and capillaries from lung of frog. Magnified 500 diameters.

Sebaceons glands in eyelid of calf, 500 diameters. The secreting cells of the gland lobules, and, their nuclei are well shown.

Section of kidney of frog, showing nuclei of secreting cells. Magnified 400 diameters.

Section of liver of Pseudo-Triton, polygonal cells and thin nuclei shown.

Section of ovary of cat, showing tissue of ovary and immature ovules imbedded, 400 diametres.

Section of ovary of cat, showing graafian follicle lined by the zona granulosu, in which is imbedded an ovule with germinal vesicle and germinal spot.

These photographs are very beautiful and show with what consumate skill Surgeon J. J. Woodward has perfected the art. The profession are placed under lasting obligations for the beautiful and truthful expressions he is furnishing both in histology and pathology.

